# Investigations of the scoring perspectives for snowboard big air based on interview and evaluation survey

**DOI:** 10.3389/fspor.2026.1793460

**Published:** 2026-04-13

**Authors:** Yurina Tsubaki, Hiroki Ozaki, Yui Kawano, Kisho Zippo, Toshiharu Yokozawa

**Affiliations:** 1 Department of Sports Sciences, Japan Institute of Sports Sciences, Tokyo, Japan; 2 Department of Sport Science, Daitoh Bunka University, Tokyo, Japan; 3 Department of Child Sports Education, Kyusyu Kyoritsu University, Fukuoka, Japan

**Keywords:** evaluation, impression, interview, judged sports, snowboard

## Abstract

**Introduction:**

Snowboard Big Air (SBA) is a judged sport evaluated on overall impression based on execution, difficulty, amplitude, variety, and progression. The specific perspectives judges use to evaluate performances and their contribution to scoring outcomes remain unclear. In this study, we investigated perspectives influencing judge evaluations in SBA.

**Methods:**

This study had a sequential exploratory mixed method design including (1) interview survey, (2) qualitative analysis and evaluation item generation, (3) capturing tricks, (4) evaluation survey, and (5) statistical analysis. Seven female athletes performed backside 900 and free trials, recorded from three video angles. Seven judges scored each trial by over and indicated their reasoning using 25 evaluation items derived from three expert judge interviews plus an “others” category. Spearman's coefficient analysis was conducted between T-scores of the overall scores and each evaluation point. Moreover, stepwise multiple regression analysis was performed to identify significant T-score predictors.

**Results:**

Correlation and regression analyses between scores and evaluation items identified 12 items significantly related to scores (|*ρ*| = 0.54–0.93, *p* < 0.05, *R²* = 0.63, *p* < 0.01). Positive items showed stronger correlations than negative items. High jumps, stable posture control, firm grabs, and smooth landings emerged as primary score predictors, whereas unstable or failed landings negatively influenced scores.

**Discussion:**

SBA scoring is a dual system: although primarily additive based on key performance factors, it also incorporates subjective, experience-driven perspectives that elude predefined metrics. This synthesis of quantifiable performance and qualitative expertise characterizes the judging process as well as underlies the inherent complexity and appeal of competitive snowboarding.

## 1 Introduction

In competitive sports, outcomes are determined through quantifiable indices such as elapsed time and distance achieved under explicitly defined rules. These measurable criteria ensure transparency, reproducibility, and consensus in performance evaluation. Conversely, judged sports, including Snowboard Big Air (SBA), figure skating, and artistic gymnastics, rely on expert appraisal of technical execution, aesthetic quality, and overall performance (1–3). Although standardized scoring guidelines exist, evaluation inherently depends on judge perspectives (4), and perspectives influencing trial scores are rarely explicit. Therefore, examining judge perspectives on trial evaluation is essential for identifying scoring criteria in judged sports.

SBA is a judged freestyle discipline where athletes perform aerial tricks after launching from a large, purpose-built jump, allowing substantial height and distance for rotations and grabs. It exemplifies the intersection of high-risk challenges, technical skill, and creative expression, combining freestyle, big-mountain riding, and individual style (5). Higher scores reward tricks demonstrating greater rotational degrees, complex off-axis movements, clean and well-held grabs, and controlled landings, whereas instability, hand drags, or falls reduce scores. Judges evaluate performances on “Execution,” “Difficulty,” “Amplitude,” “Variety,” and “Progression” using official criteria established by international governing bodies such as FIS (1). Although multiple scoring criteria exist, each judge awards one overall score (0–100 points). Given that scoring integrates quantitative and qualitative aspects, the central question is how judges interpret performance and what are their perspectives within this evaluative process.

Previous research on snowboarding performance, particularly in halfpipe and SBA, has primarily identified key biomechanical and physical parameters enabling athletes to perform complex aerial maneuvers and achieve high performance. Studies have examined take-off velocity with various tricks (6), landing stability on slopestyle jumps affected by jump design and aerial maneuvers of athletes (7), kinematic variable variability in single backside snowboard flips (8) and physiological and physical profiles (9, 10). Snow environment conditions are difficult to control, and data acquisition is often challenging. Despite limited research, valuable insights into performance demands in actual competition settings have emerged by focusing on physical and biomechanical determinants. However, few studies have addressed how judges evaluate SBA performances or which observable perspectives contribute to scoring outcomes.

Judges integrate multiple perspectives into a single evaluation decision in SBA. Clarifying judges' performance evaluation criteria would illuminate key perspectives to emphasize during actual performances. Previous works have attempted to identify factors affecting performance in judge evaluations of aesthetic disciplines, such as dance, ballet, and gymnastics (11–15). Research shows scores can vary depending on viewers, as differences in expertise, perceptual bias, or evaluative tendencies across groups may influence outcomes (14, 16–20). These results highlight the importance of methodological rigor by controlling expertise level in judging to identify performance cues and characteristics through actual evaluations by adjudicators. Consistent with this principle, in this study, we employed only internationally certified adjudicators, establishing a consistent foundation for interpreting how highly qualified adjudicators perceive and evaluate key perspectives.

We aimed to investigate the judging perspectives associated with scores in SBA through interviews and evaluation surveys from experienced judges.

## 2 Materials and methods

**Figure 1:**
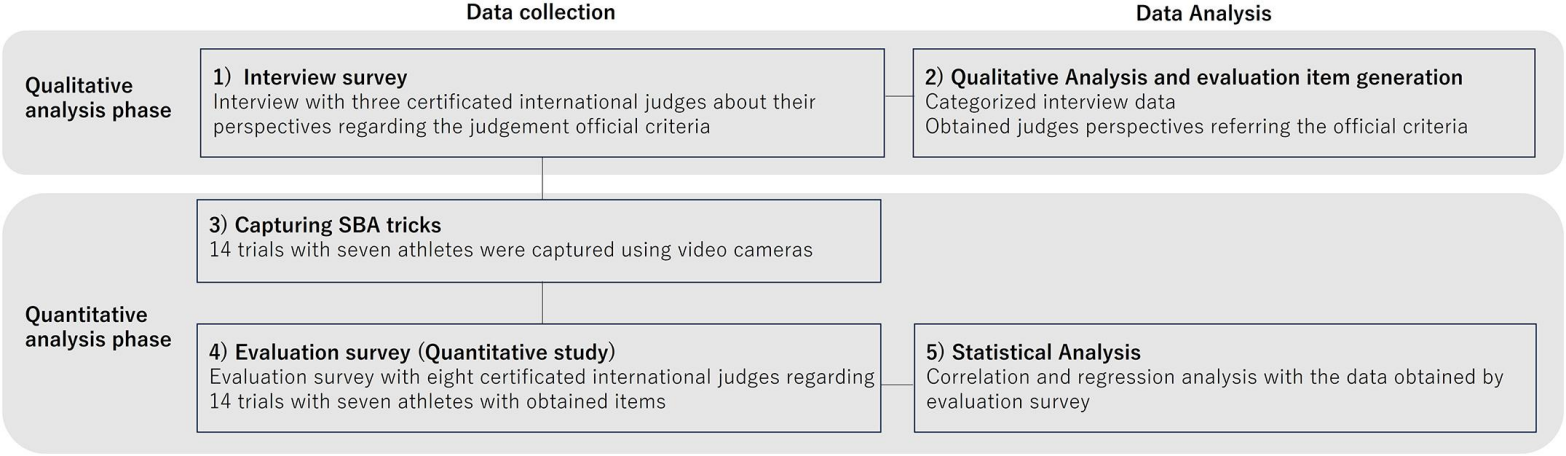
Phases of our study.

This was a sequential exploratory mixed method study. Figure 1 indicated the It comprised five steps: (1) interview survey, (2) Qualitative analysis and evaluation item generation, (3) capturing tricks, (4) evaluation survey, and (5) statistical analysis to reveal relationships between scores and judging perspective rationale. All participants signed informed consent forms after receiving detailed explanations of the study purpose, procedures, potential risks, and benefits. The study protocol received ethical approval from the Japan Institute of Sports Sciences (2023-008). All experiments were conducted in Japanese, then translated into English by the authors for reporting.

### 2.1 Interview survey

To reveal the judge's perspectives of each criterion in the official rule book, an interview survey was conducted. Three judges qualified by the International Ski and Snowboard Federation (FIS) were interviewed by a psychologist experienced in interviewing and counseling. Semi-structured interviews were conducted individually, focusing on their perspectives of criteria defined in the rulebook (1): “Execution,” “Difficulty,” “Amplitude,” “Variety,” and “Progression.” For each criteria category, judges were asked to describe what their perspectives for the criterion are during actual judging. Specifically, “execution” comprises multiple sub-elements, such as control, take-off, landing, grabs, style, course use, flow, reverts, and rails, in the official rulebook criteria, and the interviewer requested elaboration on each component. Since “course use” and “rails” were not SBA criteria, they were excluded. Interview depth varied based on interviewee responses. Each interview lasted approximately 2 h, was audio-recorded, and transcribed.

### 2.2 Qualitative analysis and evaluation item generation

To summarize the perspectives depending on each criterion, data from interview surveys were processed using a qualitative classification approach inspired by the KJ method (21). First, one researcher specializing in sports psychology who conducted interviews extracted and integrated key points related to ratings from verbatim transcripts. Subsequently, four researchers familiar with SBA characteristics, including the interviewer, held discussions to confirm and finalize classifications. After finalizing classifications, we created a list of 25 scoring items for the evaluation survey (Table 1). These items were reviewed by the lead judge to ensure that the language was comprehensible to the evaluators and then utilized as the scoring rationale in the evaluation survey. The perspectives extracted from the interviews were used as evaluation items of rationales for the scores given in the evaluation survey. This allowed quantifying the internal judging process and examining its statistical relationship with the final scores. Considering the result from the qualitative analysis, the hypothesis for the quantitative process was that judging perspectives extracted from judges' interviews are associated with scores.

**Table 1:** Evaluation items extracted from the interview survey.

Category		Evaluation items (perspectives)	Related criteria in rule book
Plus points perspective	1	Board and body rotate together during take-off	Execution (take off)
	2	Good control of body posture in the air	Execution (flow)
	3	High jump amplitude	Amplitude
	4	Attempting a difficult trick	Difficulty
	5	Trick shows originality	Execution (style) Variety, Progression
	6	Long grab duration	Execution (grabs)
	7	Good grab position	Execution (grabs)
	8	Firmly holding the board during grab	Execution (grabs)
	9	Stable landing	Execution (flow)
	10	Smooth overall motion	Execution (flow)
Impression deduction perspective	11	Knee grab	Execution (grabs)
	12	Knee lock	Execution (grabs)
	13	Fingertip grab	Execution (grabs)
Deduction perspective	14	Hand drag	Execution (landing)
	15	Edge landing	Execution (landing)
	16	Slight over- or under-rotation	Execution (reverts)
	17	Double hand touch	Execution (landing)
	18	Heavy edge landing	Execution (landing)
	19	Over- or under-rotation of >90°	Execution (reverts)
	20	Reverse rotation (landing switch unintentionally)	Execution (reverts)
	21	Unstable landing	Execution (landing)
	22	Body contact with snow	Execution (landing)
	23	Fall	Execution (landing)
	24	Complete stop after landing	Execution (landing)
	25	Landing on body before board	Execution (landing)

In the evaluation survey, “others” was added to these items.

### 2.3 Capturing SBA tricks

Seven female SBA athletes (age: 17.6 ± 3.2 years, height: 160.7 ± 2.7 cm, body weight: 56.9 ± 4.0 kg, experience: 9.3 ± 4.9 years), including three international-level athletes, performed assigned trials (Back Side 900; BS900) and preference trials on a summer jump hill. Prior to trials, athletes warmed up and practiced jumping sufficiently. In preference trials, performed tricks included CAB900, SB900, and BS1260. Capital letters indicate both rider stance and rotational direction. “CAB” (Caballerial) refers to a switch frontside spin initiated in switch stance rotating frontside, essentially a frontside spin from switch. “SB” (Switch Backside) indicates a switch backside spin, where riders take off in switch stance and initiate rotation with their back facing the spin direction. Frontside denotes spin initiated with the chest of the rider facing the rotation direction, whereas backside indicates spin initiated with the back of the rider facing that direction. Numbers represent rotation degrees (900° = two and a half spins; 1,260° = three and a half spins). Athletes wore their own clothing, helmets, goggles, and boards during trials.

**Figure 2:**
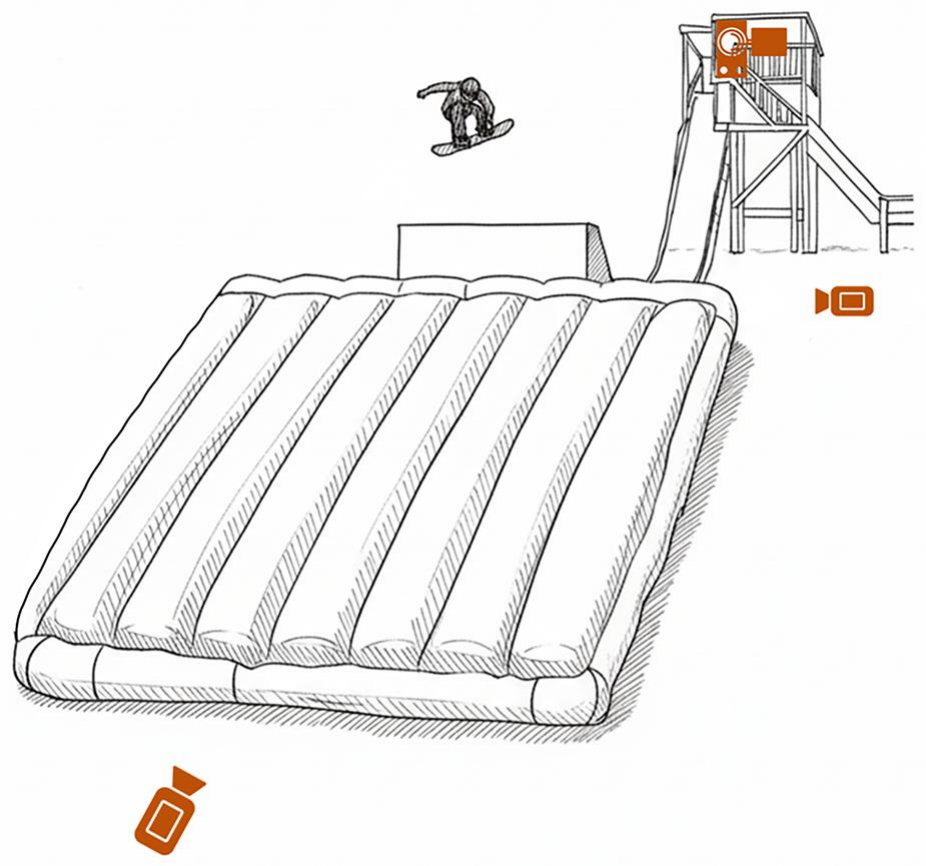
Trial environment three orange video camera icons indicate capture locations. Dimensions: lip width, 2.5 m; lip height, 2.3 m; air mat width, 73.0 m; air mat length, 21.7 m; mat thickness, 1.1 m. Distance between bottom camera and mat, 4.1 m; distance between side camera and kicker, 16.2 m.

Figure 2 illustrates the trial environment. Videos were captured from three viewpoints, top, side, and front, using video cameras at 120 fps (Sony RX0 II, Sony Corporation, Tokyo, Japan).

### 2.4 Evaluation survey

To quantify judging perspectives and examine its statistical relationship with the final scores, seven FIS-qualified judges participated in the evaluation survey, two of whom participated in the interview survey. The third interviewed judge was excluded from the evaluation survey as he typically serves as the lead judge in actual competitions and established the score range. The scoring table indicates point ranges for each difficulty level. In actual competitions, these guidelines are determined by judges before performances, primarily led by the chief judge.

The judges were seated in front of a monitor and viewed 14 trick videos (seven athletes, two trials each) presented in randomized order from three camera angles obtained during data collection. Consistent with actual competition procedures, they were permitted to watch both the original and slow-motion videos repeatedly and take notes as needed. Each judge performed assessments independently without interaction or consultation with other judges. Following standard official competition procedures, judges evaluated performances based on score ranges for each trick prepared beforehand by the lead judge. Due to the use of a highly elastic mat instead of snow, achieving stable landings was difficult, requiring judges to anticipate whether athletes successfully completed landings.

After each trial evaluation, judges first provided scores. Next, judges evaluated 26 items total, adding “others” to the 25 items in Table 1, and indicated applicable items including others. Third, if judges selected “others,” we asked for reasons, recorded voice data, and transcribed it to prevent any omission of judge perspectives regarding the scoring. This evaluation survey was conducted using Psycopy (ver 2024.2.1).

### 2.5 Statistical analysis

Final sample size was determined by methodological considerations and actual availability of judges and snowboard athletes willing to participate. Because of practical constraints such as scheduling, training and competition calendars, and recruitment feasibility in Japan, the study included all eligible individuals who could cooperate during data collection.

All statistical procedures were conducted using Python (ver. 3.13). To adjust for judges who systematically gave higher or lower scores and to equalize their evaluation influence, we calculated T-scores for judge scores:

$$\;T\;=\;50\;+\;10\;\times \frac{\left(X\;-\;M\right)}{SD}$$

where X denotes an observed score, M represents the population mean employed for standardization, and SD represents the corresponding population standard deviation. The average of seven judges' T-scores and the total number of judges who answered “applicable” for each of 25 evaluation items were used as representative values for the correlation analysis. Spearman's coefficient analysis was conducted between T-scores and each evaluation point to explore whether the perspectives obtained from the interview survey are related to the reasons for actual scoring by the qualified judges. Moreover, a stepwise multiple regression analysis was conducted to identify key predictors of T-scores. The analysis utilized each judge T-score per trial as the dependent variable, with the specific items identified by the judge during those trials serving as independent variables. To ensure the statistical integrity of the model, multicollinearity was assessed using the Variance Inflation Factor (VIF). Furthermore, the fundamental assumptions of linear regression were verified by residual diagnostics; specifically, Q-Q plots confirmed that the residuals followed a normal distribution.

For the correlation and regression analysis, the significance was set at 5%.

## 3 Results

Table 1 illustrates judge perspective categories derived from the interview survey: 10 positive scoring perspectives, 3 impression deduction perspectives, and 12 deduction perspectives.

**Figure 3:**
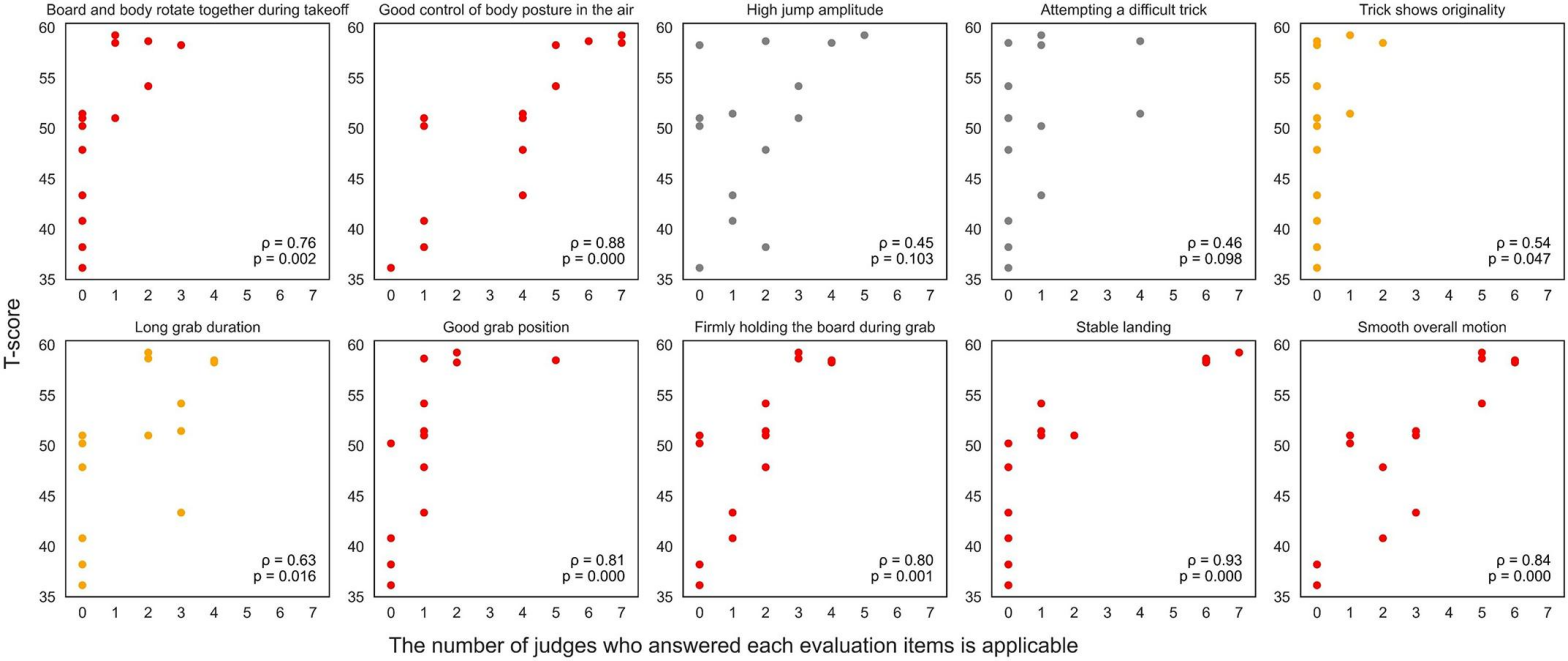
Correlation analysis between T-scores and number of judges indicating each evaluation item (positive effect) was applicable. Red and orange dots in graphs indicate significant correlations (red: *p* < 0.01, orange: *p* < 0.05).

**Figure 4:**
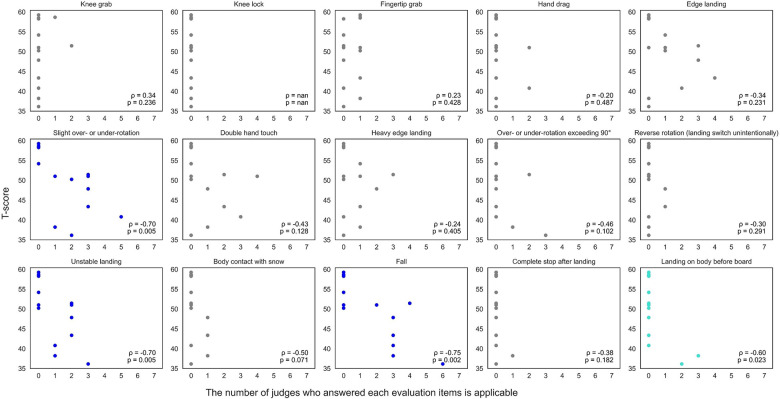
Correlation analysis between T-scores and number of judges indicating each evaluation item (negative effect) was applicable. Blue and sky-blue dots in the graph indicate significant correlations (blue: *p* < 0.01, sky blue: *p* < 0.05).

**Figure 5:**
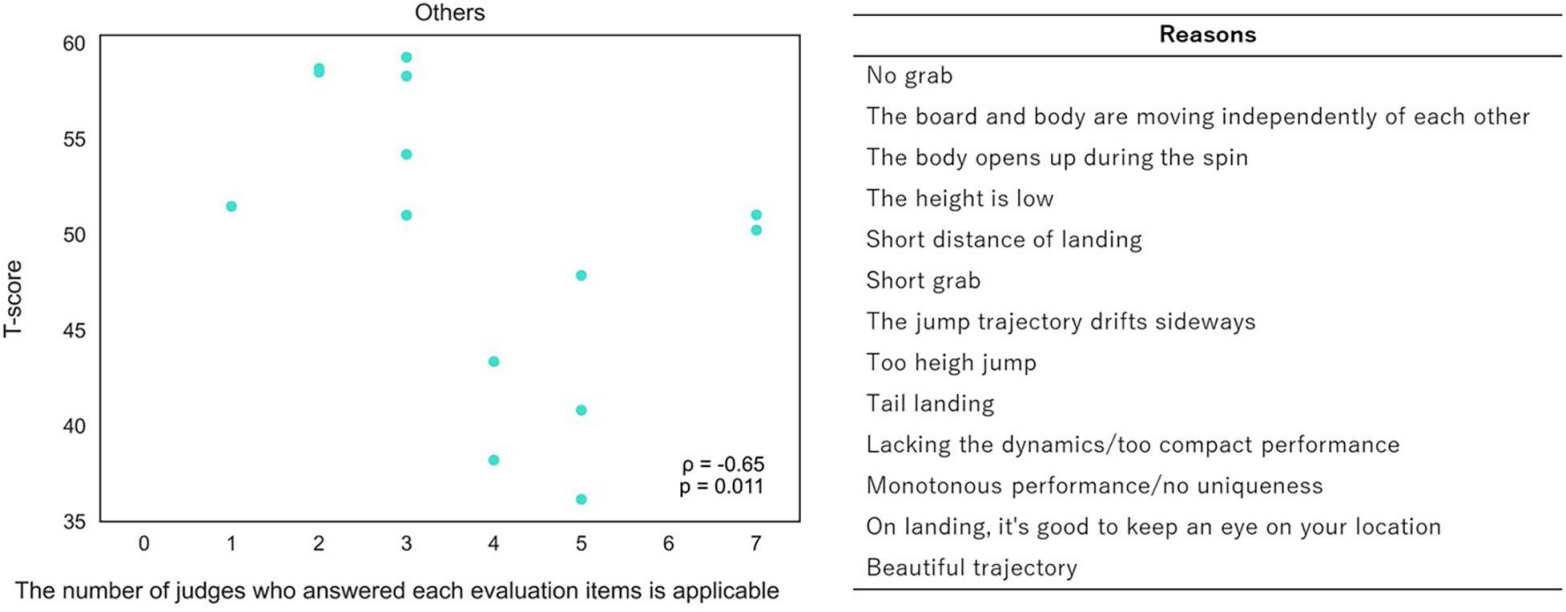
Correlation analysis between T-scores and number of judges indicating “others” (left) and table of reasons (right).

Figures 3–5 (3: positive reasons, 4: negative reasons, 5: others) show relationships between T-scores and the number of judges indicating each evaluation item was applicable across 14 trials. Thirteen evaluation items showed correlations: board and body rotating together during take-off, good body posture control in the air, trick showing originality, long grab duration, good grab position, firmly holding the board during grab, stable landing, and smooth overall motion (Figure 3: *ρ* = 0.54–0.93, *p* < 0.05), and slight over- or under-rotation, unstable landing, fall, and landing on body before board (Figure 4: *ρ* = −0.75 to −0.50, *p* < 0.05). Other evaluation items showed no correlation. Additionally, “others” had significant negative correlation (Figure 5: *ρ* = −0.65, *p* < 0.05).

Table 2 demonstrates regression analysis results (*R^2^* = 0.65, adj. *R^2^* = 0.63, *p* < 0.01). High jump amplitude, attempting a difficult trick, firmly holding the board during grab, over- or under-rotation of >90°, fall, and complete stop after landing were selected as explanatory variables.

**Table 2:** Regression analysis.

Items	*β*	*p*	VIF	95% Confidence intervals
High jump amplitude	2.97	0.028	1.11	[0.325, 5.625]
Attempting a difficult trick	5.00	0.004	1.03	[1.655, 8.350]
Firmly holding the board during grab	5.08	0.000	1.16	[2.374, 7.793]
Over- or under-rotation of >90°	−5.77	0.017	1.09	[−10.472, −1.060]
Fall	−10.80	0.000	1.16	[−13.499, −8.083]
Complete stop after landing	−12.96	0.020	1.01	[−23.800, −2.121]

(*R^2^* = 0.65, adj. *R^2^* = 0.63, *p* < 0.01).

T-score was the dependent variable; evaluation items were independent variables.

## 4 Discussion

To our knowledge, this study is the first to examine whether scoring criteria, clarified through judge interviews and evaluated via scoring surveys, influence competition results in SBA. The results indicate that although judges share a common understanding when evaluating SBA tricks, aspects remain subject to individual qualitative judgment.

Through interviews, participants described perspectives they rely on during take-off, aerial phases, grab execution, and landing, plus broader aspects such as amplitude, flow, and overall impression. We extracted 25 judgment points including posture control, grabs, and landings. These items included metrics used in quantitative analyses in prior studies (9, 22–24). This extraction process has been applied in other fields such as dance (25) and is particularly effective for comprehensively understanding perspectives contributing to success in freeride snowboarding judging by linking qualitative insights from expert athletes with quantitative performance metrics. By identifying items experienced judges considered critical, we designed better evaluation frameworks and interpreted judging criteria linking judge evaluation with qualitative criteria.

Correlation analysis indicated that judge evaluations in SBA was associated with observable indicators of control and completion, particularly in posture, grab execution, and landing quality. Items related to posture control showed consistent associations with total scores, suggesting stable body alignment and controlled rotation function as fundamental cues judges rely on when assessing technical proficiency in the air. Similarly, correlations involving grab execution highlight the importance of duration and precision, which may contribute to style. Landing performance demonstrated some of the highest correlations, reinforcing its role as a decisive judging element; stable and accurate landings provide unambiguous evidence of successful trick completion and carry substantial scoring weight. Collectively, these findings suggest judges prioritize perspectives clearly reflecting grab, control, and completion. These observable cues play central roles in differentiating performance quality under judging conditions as seen in the rulebook (1).

Furthermore, other evaluation items lacked correlation with T-scores despite extraction through interview surveys. Two possibilities explain these results. First, trials used for evaluation surveys did not include certain items, especially negative items, resulting in less evaluation deviation (e.g., knee grab, knee lock, fingertip grab, hand drag, reverse rotation, body contact with snow, complete stop after landing). Moreover, one judged item is just one element of overall impression; therefore, if another element had bigger performance impact, then that other element may have comparably small performance effect. This fact implies items are mutually interacting, as seen in regression analysis results considering items conclusively. Although we successfully identified 12 evaluation points from 25 interview survey items, “others” still appeared as judgment reasons with negative correlation (r = −0.65) despite our extracted evaluation criteria. This cannot be dismissed; additional judgment criteria beyond interview survey items may exist for each judge. This aligns with previous studies suggesting judge evaluations are influenced by observable technical cues, aesthetic perception, and individual experience, which may not be fully captured by predefined scoring items or different judge types by viewers in previous studies (14, 15, 20). Overall, correlations were observed among positive scoring items, but negative correlation was found in the “others” category. These findings suggest that although judges may seek common ground in positive scoring decisions, they may also judge performances based on individual perspectives or seek deductions that did not emerge as shared consensus from interviews. In SBA, components such as grab, trajectory, and impression may serve as additional cues contributing to scoring variability among judges as seen in the “others” answers (Figure 5). Therefore, although structured evaluation items provide systematic assessment bases, these results highlight the multidimensional nature of judging and potential influence of idiosyncratic criteria on scoring outcomes.

Moreover, regression analysis results demonstrate 63% score explanation, indicating considerable explanatory power, especially considering qualitative trick evaluation. However, this explanatory level indicates that in judged sports, performance quality cannot be fully captured by explicit criteria alone. Judges inevitably integrate sport-specific expertise, individual weightings assigned to performance perspectives, and tacit knowledge acquired through extensive experience. Judged sports character is shaped by evaluation structures where quantifiable parameters coexist with qualitative judgments. This coexistence permits creativity, individuality, and context-dependent interpretations within the sport, thereby sustainably supporting its richness and appeal. Overall, this study's findings suggest that although performance-related variables can explain significant portions of judge evaluations, remaining unexplained variation may reflect precisely these nuanced experiential dimensions making SBA complex and intriguing.

This study had some limitations. Only 14 tricks on the summer jump hill were included with insufficient variation and limited numbers of both athletes and judges. Additionally, independent variables were derived from the aggregate number of “applicable” responses for the sample size of 14 tricks. Hence, the regression analysis should be regarded as a preliminary exploration. Further research is required to verify the consistency and stability of these scoring patterns through repeated measurements. Nevertheless, this study is the first step at attempting to understand how judges evaluate tricks and reveal perspectives relating to high performance in SBA. Future studies should explore the relationships between biomechanical parameters and individual evaluation criteria for performance assessment. Such research may also help identify measurable characteristics in performances that are perceived to meet those criteria.

## 5 Conclusion

We identified high jump amplitude, attempting difficult tricks, firmly holding the board during grabs, over- or under-rotation of >90°, falls, and complete stops after landing as key performance perspectives in SBA. Positive correlations predominated across evaluated factors, indicating that SBA scoring primarily functions as an additive system. Simultaneously, negative correlations observed in the “other” category suggest that not all aspects influencing judge evaluations can be fully captured by predefined performance parameters. The remaining unexplained variance likely reflects subtle, experience-based perspectives inherent in the decision-making of judges. This coexistence of quantifiable performance characteristics and qualitative judgment appears to be a defining feature of SBA, contributing to both the complexity and enduring appeal of judged sports.

## Data Availability

The raw data supporting the conclusions of this article will be made available by the authors, without undue reservation.
